# Megalin-Mediated Trafficking of Mitochondrial Intracrines: Relevance to Signaling and Metabolism

**Published:** 2021-11-23

**Authors:** David Sheikh-Hamad, Michael Holliday, Qingtian Li

**Affiliations:** 1 Division of Nephrology and Selzman Institute for Kidney Health, Department of Medicine, Baylor College of Medicine, Houston, Texas, 77030 USA; 2 Center for Translational Research on Inflammatory Diseases (CTRID), Michael E. Debakey VAMC, Houston, Texas, 77030 USA

**Keywords:** Proteinuria, ApoE, Vitamin D, Cubulin, OCRL1, PIKfyve, Donnai Barrow and Lowe syndromes

## Abstract

The multi-ligand binding protein megalin (LRP2) is ubiquitously expressed and facilitates cell uptake of hormones, nutrients and vitamins. We have recently shown megalin is present in the mitochondria of cultured epithelial and mesenchymal cells, as well as many organs and tissues. Mitochondrial megalin associates with stanniocalcin-1 and SIRT3; two proteins that promote anti-oxidant defenses. Megalin shuttles mitochondrial intracrines (angiotensin II, stanniocalcin-1 and TGF-β) from the cell surface to the mitochondria through the retrograde early endosome to Golgi pathway and requires Rab32. Deletion of megalin impairs mitochondrial respiration and glycolysis. This pathway overlaps molecular and vesicular trafficking defects common to Donai Barrow and Lowe syndromes, suggesting that mitochondrial intracrine signaling defects may contribute to the pathogenesis of these diseases.

## Graphical abstract

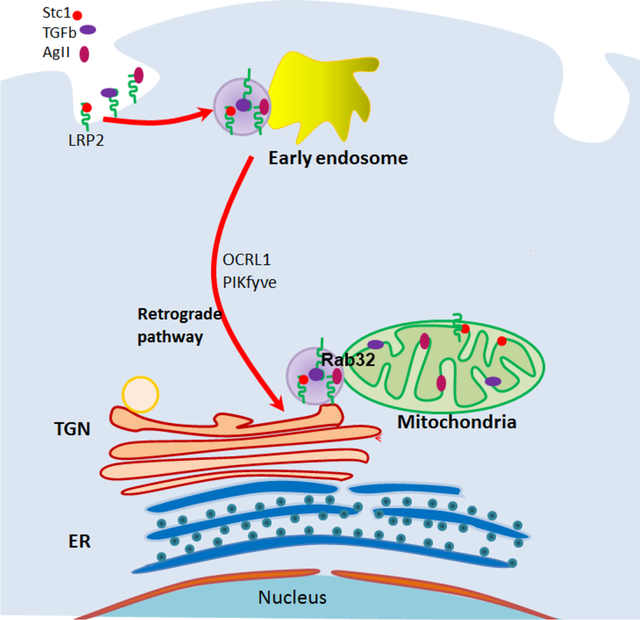

Mitochondrial intracrines (e.g., STC1, angiotensin II, TGF-b) bund to megalin at the cell surface, followed by vesicular trafficking through the retrograde early endosomes-to-Golgi pathway, and finally, Rab32-mediated fusion with the mitochondria. Stc1: Stanniocalcin1; TGFb: TGF-b; AgII: Angiotensin II; LRP2: Megalin; OCRL1: Gene mutated in Lowe Syndrome; PIKfyve: FYVE finger-containing phosphoinositide kinase (functions along OCRL1); TGN: Trans Golgi Network; ER: Endoplasmic Reticulum.

Low-molecular weight proteins, cofactors, amino acids, metabolites and many bioactive signaling molecules are filtered through the glomeruli. Evolution has yielded highly conserved pathways in proximal tubule epithelium for the reabsorption of filtered molecules; however, such absorptive capacity may be overrun, as may occur in the setting of acute and chronic glomerulopathies, or congenital and acquired proximal tubule dysfunction. Relevant to the reabsorption of filtered molecules is megalin (low density lipoprotein-receptor related protein 2; LRP2), a large, multi-ligand-binding endocytic protein with broad roles in brain development, endocrine and cytokine signaling, maintenance of physiologic hemostasis, stress response, pharmacology, and toxicology [1,2]. In the following review, we will briefly discuss some aspects of megalin’s functions and expand on the role of megalin in facilitating mitochondrial intracrine signaling (defined as extracellular signaling molecules that are targeted to the mitochondria, where they are believed to exert some of their functions [3]).

The human LRP2 gene includes 79-exons that encode a 600 kDa type I single-pass transmembrane protein [4]. The extracellular region of megalin contains cysteine-rich repeat motifs, divided into three classes: ligand-binding class A, EGF-like class B.1, and growth factor class B.2. There are 36 ligand-binding motifs, 1 EGF motif, and 15 growth factor motifs [5]. The 36 class A motifs are arranged in 4 clusters, and except for the extreme N-terminus cluster, each class A cluster is flanked by a single B.2 motif at its N-terminus and two B.2 motifs at its C-terminus [5]. The remaining 4 B.2 motifs are contained within the YWTD repeat spacer regions that separate the four class A/B.2 clusters. A single B.1 motif is located in the extracellular juxtamembrane region. The cytoplasmic tail (209 amino acids) of megalin contains two coated-pit internalization signal motifs [F(X)NPXY], several SH3 and SH2 motifs for docking of signal molecules [5], and a constitutive phosphorylation site for glycogen synthase kinase-3 (PPPSP motif), which negatively regulates megalin recycling [6]. Intramembrane proteolysis of the C-terminal fragment by gamma-secretase, yields a soluble megalin intracellular domain that may regulate proximal tubule gene expression [7,8].

Megalin is highly expressed on the luminal surface of epithelial cells such as kidney proximal tubule cells, parathyroid gland, testis, placenta, biliary system and neuroepithelium and plays critical roles in organ development and function [1,2]. Megalin is found within clathrin-coated pits in complex with cubilin and amnionless and provides receptor-mediated ligand uptake into the apical endosomal space with subsequent lysosomal targeting or transport to the basolateral cell membrane via exosome pathway [1]. Typically, hormones and vitamins (some with their accompanying binding proteins) are subjected to lysosomal degradation, with subsequent release of the ligands to the cytosol [1,9]. Embryonic and fetal brain development require megalin for appropriate epithelial tissue growth, as evidenced by the holoprosencephaly that is observed in Lrp2/megalin-deficient mice [10]. Mice with global megalin knockout die from respiratory failure due to dysfunctional type II pneumocytes [10]. Similar neurological developmental abnormalities are also observed in humans with autosomal recessive *Donnai-Barrow and facio-oculoacoustico-renal (DB/FOAR) syndrome* where various loss-of-function mutations in LRP2 result in corpus callosum agenesis, in addition to ocular, hearing, and facial abnormalities accompanied by low-molecular weight proteinuria [4]. The closely related *Lowe syndrome (oculocerebrorenal)*, an X-linked disease characterized by congenital cataracts, cognitive impairment, proximal tubular dysfunction and arthropathy, is caused by mutations in the inositol polyphosphate 5-phosphatase gene (OCRL; typically in exons 8–23) associated with impaired Rab GTPase binding and clathrin regulation leading to dysfunctional megalin-mediated endocytosis [11]. Mutations in OCRL gene (typically in exons 1–7) can also give rise to *Dent’s disease 2*, characterized by renal Fanconi syndrome with minor extrarenal manifestations [11]. LRP2 polymorphisms in various human populations have been associated with gout, diabetic nephropathy, central adiposity, gallstone disease, acute coronary syndrome, hypovitaminosis, hypercholesterolemia, Alzheimer’s disease (impaired clearance of amyloid beta peptide), neural tube defects, relapse of multiple sclerosis and intellectual disability [12–21]. A large European population study found no association between some LRP2 polymorphism and eGFR decline [22], while a study of African Americans revealed a novel missense LRP2 mutation that protected from type 2-diabetes mellitus-associated end-stage kidney disease [23].

We have recently reported the novel finding of megalin in the mitochondria [24]. In addition to the mitochondria of native kidney tubular epithelial cells and cultured cells of kidney origin (HEK293T), we detected megalin in the mitochondria of mesenchymal cells including murine macrophage-like Raw264.7 and muscle C2C12 cells [24]; mitochondrial localization of megalin is also found in cells of many tissues/organs surveyed, including the stomach, intestine, lungs, liver, spleen and hippocampus [24]. In the mitochondria, megalin exists in association with stanniocalcin-1 (Stc1) and Sirt3. These associations suggest an important role for megalin in mitochondrial functions related to these proteins. SIRT3 localizes to the inner mitochondrial membrane and matrix; it reduces ROS through nicotinamide adenine dinucleotide (NAD)^+^-dependent deacetylation of lysines on substrates involved in biosynthetic pathways such as glucose and fatty acid metabolism and the tricarboxylic acid (TCA) cycle, oxidative stress, and apoptosis [25]. STC1 is a mitochondrial intracrine [3], with binding affinity to the plasma membrane and inner mitochondrial membrane [26]. STC1 activates the metabolic sensor AMPK, which in turn upregulates mitochondrial uncoupling proteins (UCPs) and SIRT3 to promote mitochondrial anti-oxidant defenses [27]. Hypoxic preconditioning induces STC1 expression in the heart [28], and brain [29], while transgenic overexpression of STC1 confers resistance to ischemia/reperfusion (I/R) kidney injury [30]. Thus, through association with STC1 and SIRT3, megalin may play an important role in the regulation of mitochondrial anti-oxidant defenses and cytoprotection. Finally, KO of megalin in cultured cells diminishes glycolytic and respiratory capacities, suggesting that megalin is critical for mitochondrial function and cellular metabolism, either directly or indirectly [24].

Importantly, megalin is essential for shuttling of the mitochondrial intracrines STC1, angiotensin II and TGF-β from the cell surface to the mitochondria [24]. Mitochondrial intracrines include hormones (e.g., leptin, insulin, angiotensin II, angiotensin 1–7, growth hormone, atrial natriuretic peptide, prolactin, oxytocin, TRH, LGRH, VIP, somatostatin, neuropeptide Y, erythropoietin, stanniocalcin-1, etc.), growth factors (IGF-1, TGF-β, TGF-α, VEGF, PDGF, NGF, FGF1/2/3/10, etc.), cytokines/immune modulators (macrophage colony stimulating factor, INF-α, INF-β, IL-33 and other interleukins, lactoferrin, defensins) and various enzymes (urokinase, PLA2-I, PAI-2, granzyme A & B, angiotensin converting enzyme, renin/prorenin aspartyl protease, etc.) [3]. The exact function of these peptides in the mitochondria and the mechanism of their internalization and targeting to the mitochondria remain largely unknown. Based on our published work [24], internalization and targeting to the mitochondria of STC1, angiotensin II and TGF-β is megalin-dependent; shuttling is routed along the retrograde early endosome to Golgi pathway (overlaps OCRL1 and PIKfyve genes [24]; see graphical abstract), while the final leg to the mitochondria is Rab32-dependent [24]. Neither Mitoblock-6, a potent and selective inhibitor of mitochondrial Mia40/Erv1 redox-mediated import pathway [31], nor knockout of TOM40, a major subunit of the mitochondrial outer membrane translocase, inhibit trafficking of STC1 from the cell surface to the mitochondria [24]. Additionally, we found that Rab32 associates with megalin, and mitochondrial Rab32 is diminished in megalin knockout cells [24], suggesting that the function of Rab32 is intricately linked to megalin. It is important to note that the OCRL1 gene (which is mutated in *Lowe syndrome*) functions along the retrograde early endosome to Golgi pathway [32,33] and is required for normal megalin-mediated endocytosis [11]. Collectively, the data suggest that megalin and cargo (e.g., STC1, angiotensin II, TGF-β) are shuttled from the cell surface to the mitochondria through vesicular trafficking, and it is no surprise that this shuttling pathway overlaps known vesicular trafficking defects characteristic of *Lowe syndrome* (retrograde early endosome to Golgi pathway) [32]. Megalin serves as a multi-ligand receptor, and it is tempting to speculate that other known mitochondrial intracrines (beyond angiotensin II, STC1 and TGF-β, as reported by Re [3]) may be shuttled by megalin from the cell surface to the mitochondria. This insight should facilitate research into the roles/functions of other known mitochondrial intracrines [3].

While the retrograde transport pathway is well-characterized [34], only few cargo proteins are known to traffic along the retrograde pathway; these include the transferrin receptor, mannose 6-phosphate receptor, GLUT4, Atg9, EGFR, some toxins (Shiga, Cholera, Ricin) and viral particles (HIV-1 Nef and Env, AAV5) [34], raising the question of whether this elaborate and evolutionarily conserved pathway evolved to serve as a conduit for viruses and toxins. Rather, it is highly likely that this pathway serves fundamental biological processes and is being subverted by toxins and viruses for entry into the cell. Because of commonalities between the mitochondrial intracrine shuttling pathway (*megalin; retrograde early endosome to Golgi pathway*) and molecular/trafficking defects characteristic of Donnai-Barrow (*megalin mutations*) and Lowe (*OCRL gene mutations, and trafficking defects along the retrograde early endosome to Golgi pathway*) syndromes, we propose that: A) the retrograde early endosome-to-Golgi pathway may in fact serve as a mitochondrial intracrine signaling route; B) the pathogenesis of *Donnai-Barrow and Lowe syndromes* may be linked to defects in mitochondrial intracrine signaling/function.

As mentioned above, a number of cytokines and modifiers of inflammation are among the mitochondrial intracrines [3], suggesting that cytokines may play a role in regulating mitochondrial functions. Similarly, megalin has been implicated directly or indirectly in regulating inflammation and immunity. *In vivo* and *in vitro* studies suggest an important role for megalin as a transducer of tubulointerstitial nephritis in a model of protein overload [35]. Rats loaded with albumin exhibited increased megalin expression, and megalin silencing resulted in inhibition of IL-1β, IL-18 and cleaved caspase-1 [36]. IL-4 appears to provide protection from protein overload-mediated (megalin-facilitated) tubulointerstitial injury, and knockout of interleukin-4 resulted in decreased megalin expression and higher proteinuria [37]. These findings suggest a dynamic relationship between proximal tubule cell megalin and inflammation in proteinuric renal disease. In addition to the interaction with cytokines, megalin/cubulin are responsible for the uptake of filtered nephrotoxins (e.g., aminoglycoside antibiotics, cisplatin, etc.); in doing so, they play critical roles in the development of certain types of acute kidney injury [38]. Conversely, megalin/cubulin also mediate the uptake of vitamins/nutrients (e.g., retinol, B12, vitamin D, lipoproteins, etc.) and cytoprotective molecules (e.g., selenoproteins, defensins, etc.) [38]. Thus, modulation of megalin/cubulin expression may be an important component of cell adaption to injury, as well as protection from- and regeneration after injury [38]. In the remaining paragraphs, we will focus on few mitochondrial intracrines because of the unique opportunities to further elucidate their roles in mitochondrial function; these include, vit D, components of the renin/angiotensin system and sex hormones.

## Vitamin D

Hydroxylation of vitamin D to the activated form occurs to a large extent within the proximal tubule, in a manner that is dependent on megalin/cubilin-mediated endocytosis [39]. Nykjaer et al. reported in a study of megalin-knockout mice that the few mice surviving to adulthood exhibited severe vitamin D deficiency and bone disease due to failure in proximal tubule reabsorption of 25-(OH) vitamin D and its binding protein [40]. Similarly, mice with kidney tubular epithelium knockout of megalin develop hypocalcemia and osteomalacia due to lack of vitamin D [41]. The role of megalin-mediated vitamin D uptake was subsequently demonstrated *in vitro* in a human proximal tubule model [42]. Given that 25-hydroxy-vitamin D-1α-hydroxylase (CYP27B1, P450C1-α) is a mitochondrial enzyme [43], we propose that megalin may shuttle 25-hydroxy-vitamin D with its binding protein to the mitochondria, where the 1α hydroxylation of 25-hydroxy-vitamin D occurs. Vitamin D receptor (VDR) is found in the mitochondria as well, and recent evidence suggests that vitamin D receptor regulates mitochondrial respiration and ATP generation in C2C12 myoblasts [44]. Changes in LRP2 expression across various types of human nephropathy are heterogeneous [45], but given the conclusive role for megalin in vitamin D uptake, disease specific changes in megalin expression likely impact mineral-bone metabolism.

## Renin-angiotensin system (RAS)

The renin-angiotensin system regulates multiple physiological functions through angiotensin II type 1 and type 2 receptors. Both receptors, as well as angiotensin II are present in the mitochondria [46]. Activation of the mitochondrial angiotensin system is coupled to mitochondrial nitric oxide production and can modulate respiration [46]. Inhibition of megalin expression in mouse kidney reduced angiotensinogen and renin uptake in kidney proximal tubule, and increased urinary renin and angiotensinogen, establishing a role for megalin in regulating the intrarenal renin-angiotensin system [47]. As a human correlate, renin and angiotensinogen are also increased in the urine of patients with Lowe syndrome and Dent’s disease [47].

## Select hormones

Megalin mediates endocytosis of sex hormone (in complex with sex hormone binding protein) in reproductive organs such as testis and ovary; mice with megalin knockout demonstrated failure of sexual development [48]. Vitamin A with retinol binding protein, as well as transcobalamin/vitamin B12 complexes, are transported intracellularly via endocytosis by megalin [49]. Kidney-specific knockout of megalin in mice resulted in loss of retinol in the urine, indicating a role for megalin in maintaining systemic vitamin A levels [50].

In summary, megalin shuttles signaling molecules from the cells surface to the mitochondria via the retrograde early endosomes-to-Golgi pathway and Rab32. Megalin knockout impairs mitochondrial respiration and glycolysis. These findings provide insight into previously unknown functions for megalin and provide new opportunities for research into the actions of megalin-mediated hormonal shuttle to the mitochondria.
